# An Rb1-dependent amplification loop between Ets1 and Zeb1 is evident in thymocyte differentiation and invasive lung adenocarcinoma

**DOI:** 10.1186/s12867-015-0038-4

**Published:** 2015-03-19

**Authors:** Kevin C Dean, Li Huang, Yao Chen, Xiaoqin Lu, Yongqing Liu

**Affiliations:** Department of Ophthalmology and Visual Sciences, University of Louisville Health Sciences Center, 301 E. Muhammad Ali Blvd., Louisville, KY 40202 USA; James Graham Brown Cancer Center, University of Louisville Health Sciences Center, Louisville, KY 40202 USA; Birth Defects Center, University of Louisville Health Sciences Center, Louisville, KY 40202 USA; College of Agriculture and Biotechnology, Zhejiang University, Hangzhou, Zhejiang Province 310058 China; The Second Affiliated Hospital, Central South University Xiangya School of Medicine, Changsha, Hunan Province 410011 China

**Keywords:** Ets1, Zeb1, miR-200, Thymocyte differentiation, Lung adenocarcinoma

## Abstract

**Background:**

Ras pathway mutation leads to induction and Erk phosphorylation and activation of the Ets1 transcription factor. Ets1 in turn induces cyclin E and cyclin dependent kinase (cdk) 2 to drive cell cycle progression. Ets1 also induces expression of the epithelial-mesenchymal transition (EMT) transcription factor Zeb1, and thereby links Ras mutation to EMT, which is thought to drive tumor invasion. Ras pathway mutations are detected by the Rb1 tumor suppression pathway, and mutation or inactivation of the Rb1 pathway is required for EMT.

**Results:**

We examined linkage between Rb1, Ets1 and Zeb1. We found that an Rb1-E2F complex binds the *Ets1* promoter and constitutively limits Ets1 expression. But, Rb1 repression of Zeb1 provides the major impact of Rb1 on Ets1 expression. We link Rb1 repression of Zeb1 to induction of miR-200 family members, which in turn target Ets1 mRNA. These findings suggest that Ets1 and Zeb1 comprise an amplification loop that is dependent upon miR-200 and regulated by Rb1. Thus, induction of Ets1 when the Rb1 pathway is lost may contribute to deregulated cell cycle progression through Ets1 induction of cyclin E and cdk2. Consistent with such an amplification loop, we correlate expression of Ets1 and Zeb1 in mouse and human lung adenocarcinoma. In addition we demonstrate that Ets1 expression in thymocytes is also dependent upon Zeb1.

**Conclusions:**

Taken together, our results provide evidence of an Rb1-dependent Ets1-Zeb1 amplification loop in thymocyte differentiation and tumor invasion.

**Electronic supplementary material:**

The online version of this article (doi:10.1186/s12867-015-0038-4) contains supplementary material, which is available to authorized users.

## Background

c-Ets1 was identified as a proto-oncogene based on v-ets in the genome of the avian leukemia retrovirus E26, and is the founding member of the Ets family of transcription factors [1]. Elevated Ets1 expression has been observed in many invasive and metastatic solid tumors [2] and Over-expression of Ets1 is sufficient for transformation of NIH3T3 fibroblasts [3]. In adults, expression of Ets1 becomes restricted primarily to lymphoid tissues [4], and mutation of *Ets1* in mice leads to defects in maturation of lymphocytes [5-7]. Ets1 interacts with Tlx to cause the critical maturation arrest in T cell acute lymphoblastic leukemia [8]. Induction of Ets1 in solid tumors triggers neovascularization and the epithelial-mesenchymal transition (EMT) that drives tumor invasion [9,10]. Ras pathway signaling is critical for normal development, and constitutively activating Ras mutations in tumors short-circuit the pathway leading to growth factor-independent cell proliferation, neovascularization and EMT [11,12]. Ets1 is phosphorylated and activated by Erk phosphorylation when the Ras pathway is engaged [13-15], and this induction of Ets1 is a mediator of Ras-initiated EMT. Accordingly, a downstream target of Ets1 is the EMT transcription factor Zeb1 [16], which is required for maintaining epithelial vs. mesenchymal balance in vivo [9]. When induced in response to Ras mutation, Zeb1 causes transition to an invasive mesenchymal phenotype [17]. A key sensor of mutant Ras is the Rb1 family of cell cycle regulators, whose activation in response to Ras mutation represses Zeb1 and blocks EMT [18].

Recent studies have found that Ets is repressed by miR-200 family members [19]. miR-200 also represses Zeb1, but in a double negative loop Zeb1 binds the promoters of miR-200 family members and represses their expression [20,21]. Such findings raised the possibility that Zeb1 might feedback to induce Ets1 via its repression of miR-200, and that Rb1 might also influence Ets1 expression via its regulation of Zeb1. Here, we examined potential linkage between Rb1, Ets1, and Zeb1. Although Rb1 can interact with genes in a cell cycle-dependent fashion to regulate proliferation, it is also found constitutively at other genes including pro-apoptotic factors and mutation or inactivate of Rb1 is required for induction of such genes [22]. We found here that Rb1 is present constitutively at the Ets1 promoter and removal of an Rb1-E2F complex using a dominant negative-E2F led to induction of Ets1. Thus, Rb1 directly diminishes the level of Ets1 expression. We also provide evidence that Zeb1 induces Ets1, and we show that an additional and major effect of Rb1 on Ets1 expression is mediated through Rb repression of Zeb1. We link the effect of Zeb1 to its regulation of miR-200, which in turn target Ets1.

Taken together, our results provide evidence of an amplification loop consisting of Ets1 and Zeb1, which is mediated by miR-200 and regulated by Rb1. We also show that Zeb1 and Ets1 are expressed together at the invasive edge of K-Ras-initiated mouse lung adenocarcinomas, and there is a significant correlation between expression of Ets1 and Zeb1 in human lung adenocarcinoma. Like Ets1, Zeb1 is important for thymocyte differentiation, and *Zeb1*(−/−) mice show a reduction in thymocytes [5,23-27]. Importantly, we demonstrate that mutation of *Zeb1* eliminates Ets1 expression in thymocytes, demonstrating dependence of Ets1 expression on Zeb1 in thymocytes, and thus potentially linking the Zeb1 phenotype in T cell differentiation to a lack of Ets1 expression.

## Methods

### Cells and cell culture

Rb family triple knockout (TKO) mouse embryo fibroblasts and control wild-type fibroblasts have been described and were a kind gift from T. Jacks and J. Sage [28]. Three independent TKO and wild-type isolates were used with similar results. Mouse Zeb1 wild type and mutant fibroblasts were isolated from crosses of mice heterozygous for Zeb1 and genotyped as described [23]. The human osteosarcoma U2OS cells expressing IPTG-inducible p16^INK4a^ were described previously [29], as were the U2OS cells expressing both IPTG-inducible p16^INK4a^ and DN-E2F-mER [30]. U2OS cells were cultured with 1 mM IPTG in the medium for either one or three days to induce p16^INK4a^, or with 100 nM Tamoxifen (OHT) for one day to induce mER-DB-E2F expression. For combined treatments, cells were treated with IPTG for one day, and then OHT was added along with the IPTG for an additional day. All the above fibroblast cells were cultured in DMEM medium with 10% fetal bovine serum (FBS) and antibiotics at 10% CO_2_ and 37°C.

### Zeb1 shRNA construct and lentivirus preparation

We have described the lentiviral shRNA knockdown of Zeb1 protein and mRNA in detail previously [17,18,31,32,33].

### Immunohistochemistry

Mouse embryos and adult lung tumor tissues were fixed with 10% formalin, paraffin-embedded, and sectioned at 5 μm. Primary antibodies for Zeb1 (kind gift from Dr. Douglas Darling), CD3 (kind gift from Dr. Qingxian Lu), E-cadherin (Santa Cruz Technology), Ets1 (Santa Cruz Biotechnology) and the secondary antibodies are detailed in Additional file 1: Table S1. The slides were mounted with coverslips using anti-fade medium Permount with DAPI (Fisher) and viewed under a Zeiss fluorescent microscope.

### K-Ras^LA1^ mice

Housing and handling of all mice was in accordance with procedures approved by the University of Louisville Institutional Animal Care and Use Committee (IACUC). K-Ras^LA1^ mice [34] in a C57BL6 background were obtained from Jackson Laboratory. PCR genotyping was as described previously [34].

### Human lung adenocarcinoma microarray analysis

No human tissue was utilized in this study. The processed microarray data were used from previously published work (the NCBI database GSE_1969163) that contains 59 samples of human lung adenocarcinomas sequenced for mutations in K-RAS and EGFR and patient matched control lung tissue [35]. The expression levels of Zeb1 and Est1 mRNAs were selected for calculating Pearson correlation score.

### Chromatin immunoprecipitation (ChIP) assay

ChIP assays were based on the UpState protocol (http://www.tc.umn.edu/~muell002/Laboratory/protocols/X-ChIP.htm) using formaldehyde to crosslink genomic DNA of wild-type MEF cells. The chromatin was sheared to an average length of 300–500 bp. Monoclonal antibodies for Rb (Santa Cruz sc-50), E2F1 (Santa Cruz sc-193), E2F4 (Santa Cruz sc-866), and polyclonal antiserum for Zeb1 (a kind gift from Dr. Douglas Darling) were used for immunoprecipitation. Equal amounts of anti-IgG or pre-immune serum were used as controls. The sequence of primers for *Ets1* promoter and the expected size of the PCR products are show in Additional file 2: Table S2. ChIP PCR reactions were similar to those described below for real-time PCR, but with additional 1% BSA and 1% DMSO, and the PCR programs usually had a higher annealing temperature (e.g. 60 – 68°C) and longer extension time (e.g. 1 minute).

### Protein extraction and electrophoretic mobility shift assay (EMSA)

The cultured adherent wild-type MEF cells were scraped off and lysed in the lysis buffer (50 mM Tris/HCl, pH7.4, 100 mM NaCl, 0.5% Triton X-100, 0.5% NP-40) on ice for 20 minutes, followed by 10-minute centrifuge at 13,000 g, the supernatant was thereafter collected as non-denatured protein extract immediately for the gel shift assay. Based on the sequence alignment of both human and mouse *Ets1* promoters, an aligned 24-bp short sequence (5′-TCCATAATTTGCCACTGATAGAT-3′) with a putative E2F site (TTTGCCAC) is selected for synthesis of the double-strand oligos with and without artificial mutation of the E2F site (the mutant oligo sequence: 5′-TCCATAACCTAGCTAGATAGAT-3′). Twenty μg of the above crude protein lysate was mixed with 10 pmol of each of the double-strand oligos in a 10-μl protein-DNA binding buffer (5 mM Tris/HCl pH7.4, 100 mM KCl, 100 μM dithiothrreitol, 100 μM EDTA), and incubated at RT for 40 minutes. The binding reactions without protein lysate or oligos were served as background controls. All the binding reaction samples were separated on a 6% native polyacrylamide gel in 0.5× TBE buffer at constant 200 V and RT for 20 minutes. The finished gel was stained with 10,000× diluted SYBR green (Molecular Probe) in 0.5× TBE buffer at RT for 20 minutes, and then visualized under UV light.

### RNA extraction and real-time PCR

Total cellular RNA was extracted using TRIzol solution (Invitrogen, Carlsbad, California). Using Primer3 (http://bioinfo.ut.ee/primer3-0.4.0/), primer sets were designed to generate 150–250 base pair PCR products that bridge two separate exons. Primer sequence, melting temperature (Tm), and PCR product sizes are listed in Additional file 2: Table S2. First-strand complementary DNA (cDNA) synthesis was carried out in 20-μl reactions containing 1-2 μg of total RNA, 500 ng random hexamers, 10 mM dithiothreitol, 500 μM dNTP mix, 40 U RNaseOUT™ ribonuclease inhibitor, and 200 U M-MLV reverse transcriptase, at 37°C for 1 h according to the manufacturer’s protocol (Invitrogen, Carlsbad, California). Real-time quantitative PCR was performed in 25-μl reaction volumes containing 0.25-μl aliquots of cDNA, gene-specific primer pairs, and SYBR Green I fluorescent dye (Molecular Probes, Eugene, Oregon), in an Mx3000P Real-Time PCR System (Stratagene, Cedar Creek, Texas), according to the manufacturer’s instructions. The PCR cycle parameters were set at 95°C for 20 sec, 60°C for 30 sec, and 72°C for 30 sec, for a total of not more than 45 cycles. The fluorescent intensity of SYBR green was monitored at the end of each extension step; relative amounts of the target cDNA was estimated by the threshold cycle (Ct) number, and normalized to two internal control genes, β-actin (ACTB) and glyceraldehyde-3-phosphate dehydrogenase (GAPDH). Three independent samples were analyzed for each condition and/or cell type, and each sample was compared in at least 3 independent RT-PCR amplifications.

### MicroRNA detection

The quantitative RT-PCR to detect miRNAs is as described [36]. Briefly, polyadenylation of at least 5 μg of the total RNA was completed by poly(A) polymerase kit (PAP, Ambion) in a 20 μl of reaction volume according to manufacturer’s instruction. The polyadenylated RNA was thereafter directly utilized for cDNA preparation using a reverse transcription kit (M-MLV reverse transcriptase, Invitrogen) and an adaptor primer (5′-GCGAGCACAGAATTAATACGACTCACTATAGG(T) _12_VN*-3′) in a 40 μl of reaction volume. Real-time quantitative PCR was performed using a universal primer (5′-GCGAGCACAGAATTAATACGAC-3′) and a miRNA-specific primer (Additional file 2: Table S2) as described above.

## Results

### The Rb1 family represses Ets1 expression

**Figure 1:**
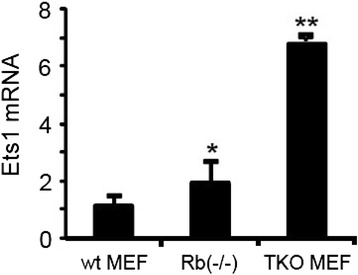
**Ets1 is under repression by the Rb1 family.** Mutation of *Rb1* in primary cultures of mouse embryo fibroblasts (MEFs) led to significant (*p* < 0.05) induction of Ets1 mRNA, and Ets1 mRNA was further very significantly (*p* < 0.01) induced when all three closely related Rb1 family members (Rb1, Rbl1 and Rbl2) were mutated to generate triple knockout (TKO) MEFs. Real time PCR results normalized to actin mRNA are shown.

Mutations in Ras trigger activation of Rb1 and tumor suppressing oncogene-induced senescence [17,37]. Thus, mutation or inactivation of the Rb1 pathway, which is a hallmark of cancers, is critical for Ras-initiated tumor formation. Ets1 is phosphorylated and activated by Erk in response to Ras signaling [13], and in turn Ets1 induces cyclin E and cdk2 to drive cell cycle progression [38]. Therefore, we wondered if Rb1 might repress Ets1. Indeed, mutation of Rb1 in primary cultures of mouse embryo fibroblasts (MEFs) led to induction of Ets1, and Ets1 was further induced when all three closely related Rb1 family members (Rb1, Rbl1 and Rbl2) were mutated to generate triple knockout (TKO) MEFs (Figure 1). These results suggest that Ets1 is under repression by the Rb1 family.

### E2F1-Rb1 binds the Ets1 promoter and constitutively repressed the gene

**Figure 2:**
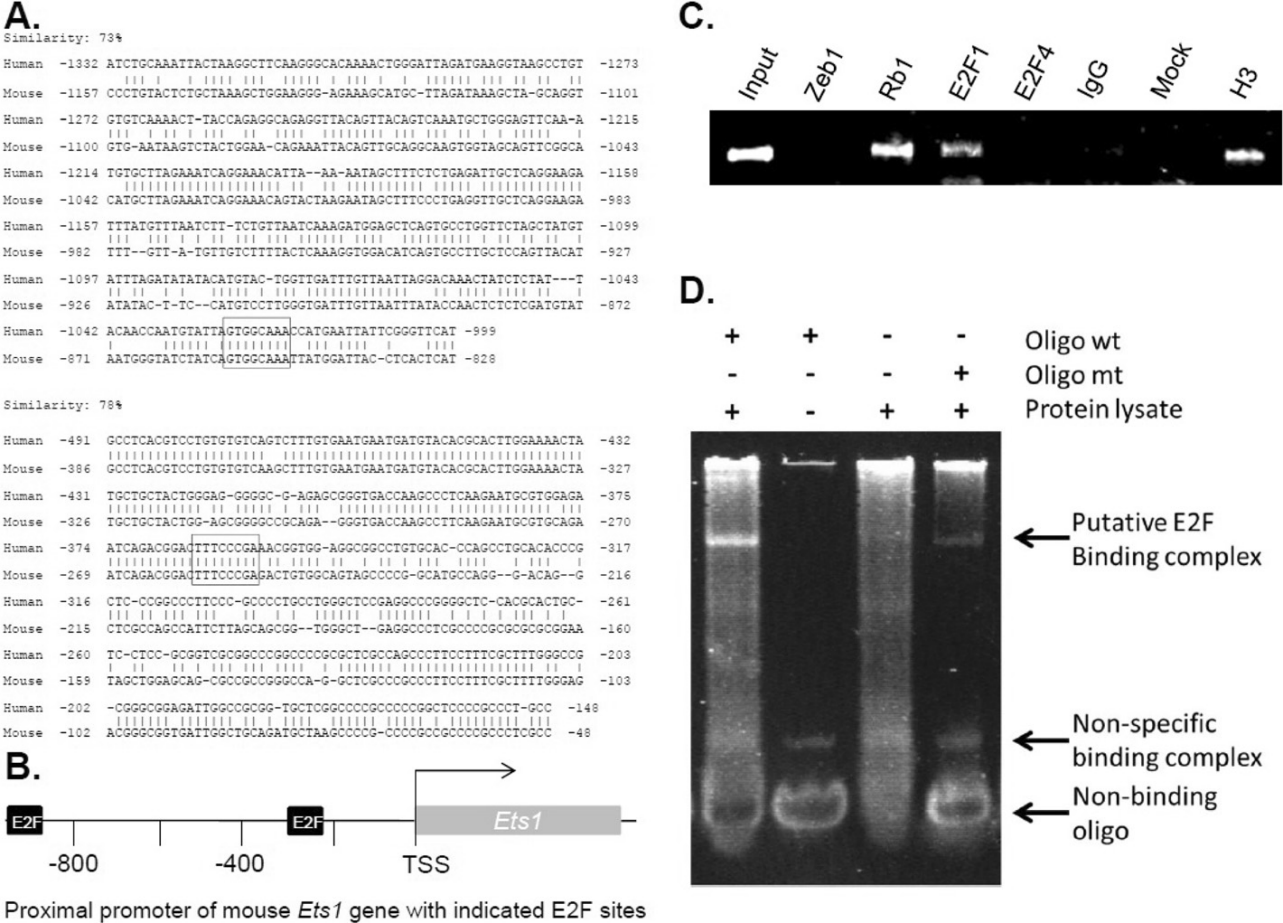
**An Rb1-E2F1 complex binds the** ***Ets1*** **promoter and constitutively represses the gene. (A)** Alignment of both human and mouse *Ets1* promoter sequences showing two regions of higher similarity that contains consensus E2F binding sites (boxed). **(B)** A schematic diagram of mouse *Ets1* promoter with two putative E2F sites. SST: start site of transcription. **(C)** Chromatin immunoprecipitation (ChIP) assay demonstrates binding of Rb1 and E2F1 to the *Ets1* promoter together with the positive control histone H3. Input indicates the DNA used for immunoprecipitation, IgG is control serum and mock indicates no added DNA. **(D)** Electrophoretic mobility shift assay (EMSA) demonstrates reduction of the specific binding of the crude protein lysate to the target DNA oligo upon mutation of its putative E2F site. ‘wt’ denotes a wild-type oligo with a putative E2F site while ‘mt’ indicates a mutant oligo with a mutated E2F site.

**Figure 3:**
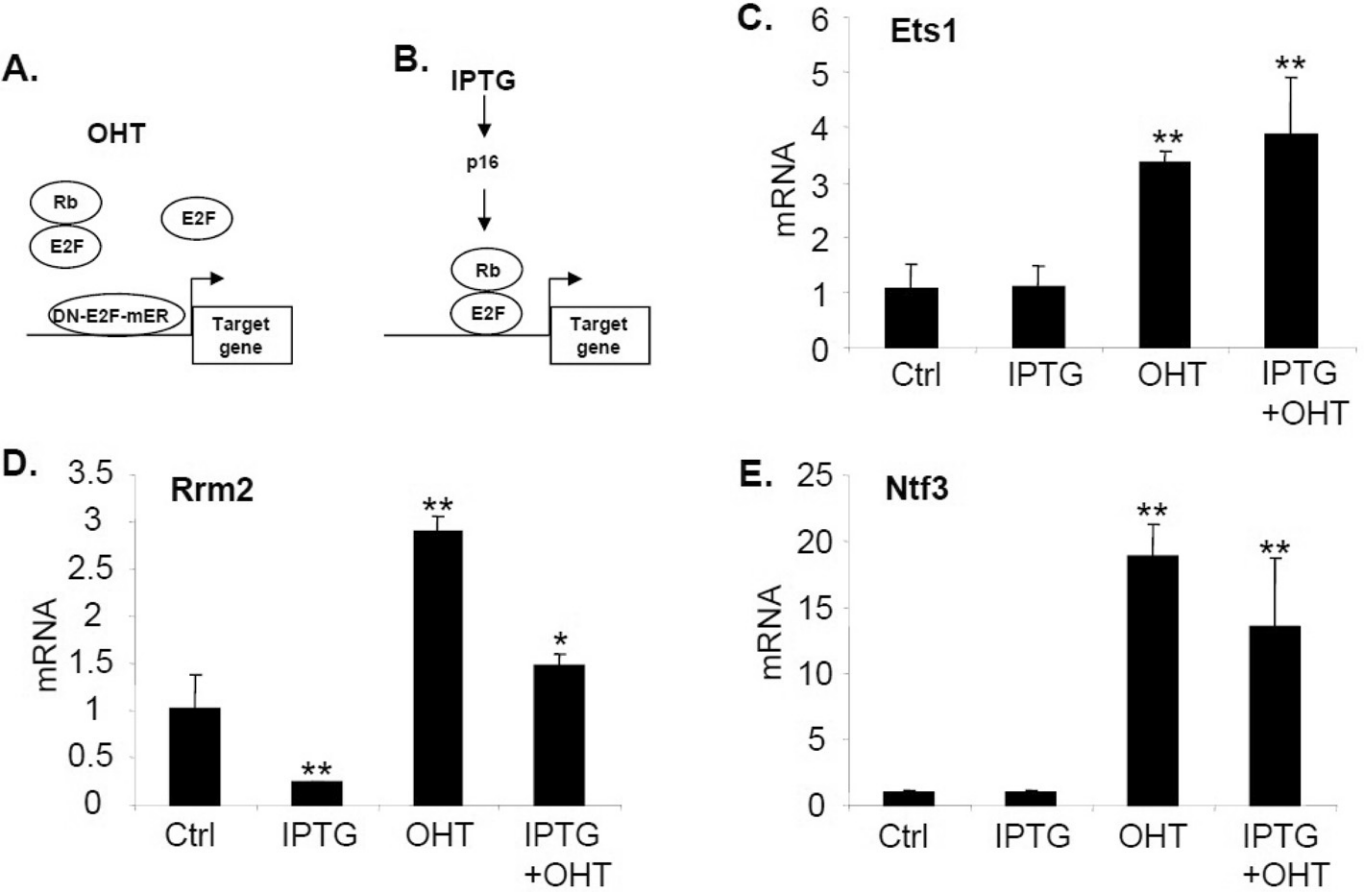
**Regulation of Ets1, Rrm2 and Ntf3 expression by Rb-E2F complex. (A)** Diagram showing a dominant negative (DN) form of E2F where the E2F DNA binding domain is fused to the mutant hydroxyl-tamoxifen (OHT)-dependent estrogen receptor (m-ER) displacing E2F and Rb-E2F from a promoter following OHT treatment. IPTG-inducible p16 expression is shown leading to assembly of E2F-Rb complexes at target genes [40]. **(B)** Human U2OS cells were treated with IPTG, OHT or IPTG + OHT as described in Material and Methods. Real time PCR was used to measure mRNA levels of Ets1 **(C)**, Rrm2 **(D)** and Ntf3 **(E)**. “Ctrl” indicates untreated control cells. Results were normalized to β-actin mRNA. Rrm2, ribonucleotide reductase, Ntf3, neurotrophin 3.

We used chromatin immunoprecipitation (ChIP) assay and electrophoretic mobility shift assay (EMSA) to determine whether Rb1 is bound to the *Ets1* promoter and whether removal of its putative binding site of E2F family, the Rb1 DNA binding partner, diminishes the binding. Rb1 does not bind directly to DNA, but it can be tethered to promoters through interaction with the E2F family of transcription factors [39]. Some E2Fs (e.g. E2F1) contain a transactivation domain, while other E2Fs lack a transactivation domain (e.g., E2F4). Binding to Rb1 blocks the transactivation domain of E2Fs, but Rb1 also interacts with epigenetic modifiers to form an active repressor [29]. Both human and mouse *Ets1* promoters have two major similar regions that each contains a perfectly aligned consensus E2F binding site (Figure 2A and B). ChIP assays showed that Rb1 and E2F1, but not E2F4, were bound to the *Ets1* promoter in vivo (Figure 2C), while EMSA demonstrated that mutation of the putative E2F site reduced the specific binding of the crude protein lysate to the target DNA oligo in vitro (Figure 2D), suggesting that Rb1-E2F1 complex specifically binds to the putative E2F site of the *Ets1* promoter. Next, we wondered if Rb1 was acting at the *Ets1* promoter to block transactivation by E2F, or if Rb1 was serving as an active repressor. Previously we described a dominant negative (DN) form of E2F consisting of the DNA binding domain fused to a mutant estrogen receptor (DN-E2F-mER). Following hydroxyl-tamoxifen (OHT) treatment, DN-E2F-mER is translocated to the nucleus where it displaces E2F and E2F-Rb1 complexes from promoters. In the same cell, an IPTG-inducible *p16* gene is also included to assemble E2F-Rb complexes at target genes following IPTG treatment [40] (Figure 2A). If E2F were serving a transactivation function at the *Ets1* promoter, then DN-E2F-mER would cause repression, but if an E2F-Rb1 complex were serving as a constitutive repressor, DN-E2F-mER would cause induction. We found that following tamoxifen treatment, DN-E2F-mER caused induction of Ets1, demonstrating that E2F1-Rb1 at the promoter is constitutively repressing *Ets1* (Figure 3C). Rb1 activity is regulated in a cell cycle dependent fashion by cyclin dependent kinases (cdks) that hyperphosphorylate the protein blocking its interaction with E2F [22]. Cdk inhibitors such as p16 block the activity of these cdks and cause accumulation of constitutively active Rb1 which associates with E2F at the promoter of target genes [41]. Many genes such as *Rrm2* are dependent upon phosphorylation/dephosphorylation of Rb1 during the cell cycle and these genes are further repressed when Rb1 is activated by expression of p16 (Figure 3D), but other genes such as *Ntf3* that are constitutively repressed by Rb1-E2F are not further repressed by activation of Rb1 through expression of p16 (Figure 3E). We previously described expression of p16 driven by an IPTG-inducible promoter [32] (Figure 3B). As with Ntf3, we found that induction of p16 had no effect on repression of Ets1 (Figure 3C). Taken together, these results suggest that E2F-Rb1 is present constitutively at the Ets1 promoter, where it reduces the level of Ets1 expression in a cell cycle-independent fashion.

### Zeb1 repression of the miR-200 family is linked to induction of Ets1

**Figure 4:**
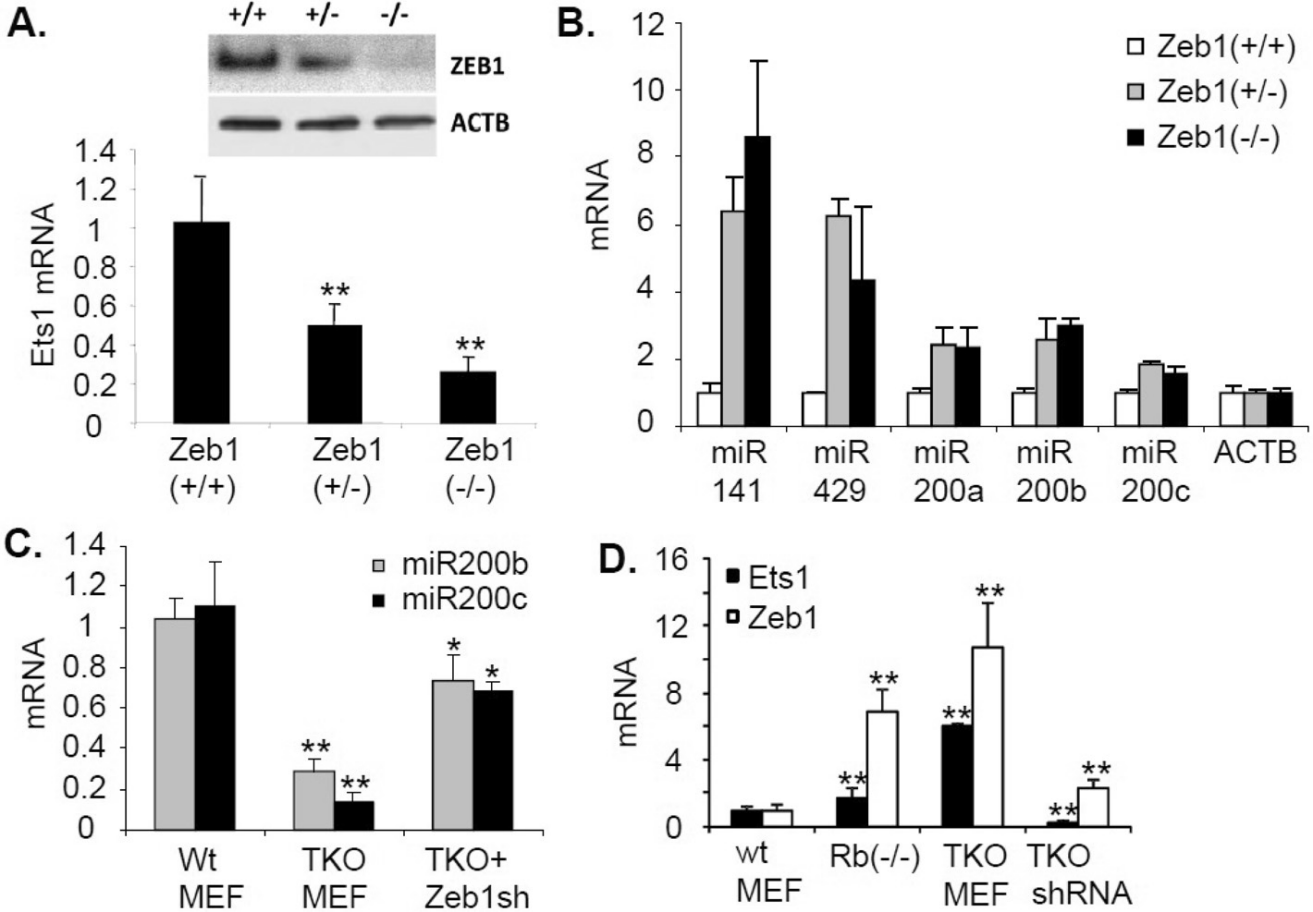
**Zeb1 induces Ets1 expression, and it mediates much of the effect of Rb1 on Ets1 expression. (A)** *Zeb1* mutation leads to a gene dosage-dependent downregulation of Ets1 expression. Real time PCR is shown and results are normalizaed to β-actin mRNA when the Western blots show similar result accordingly. **(B)** *Zeb1* mutation leads to a gene dosage-dependent induction of miR-200 family members. Real time PCR is shown. **(C)** *Rb1* family mutation leads to induction of miR-200 family members, and this induction is reversed by lentiviral knockdown of Zeb1 (Zeb1 sh). **(D)** Zeb1 is induced along with Ets1 as *Rb1* family members are mutated. And, knockdown of Zeb1 reverses most of the induction of Ets1. Real time PCR is shown.

Zeb1 is a target of the miR-200 family, but in a negative loop Zeb1 feeds back to repress transcription of miR-200 family members [20,21]. Because miR-200 has been shown to target Ets1 [19], we wondered if Zeb1 might also affect expression of Ets1. Indeed, we found that mutation of *Zeb1* in MEFs led to a gene dosage-dependent increase in expression of Ets1 (Figure 4A). We did not detect Zeb1 at the *Ets1* promoter in ChIP assays (Figure 2C) and induction of Ets1 coincided with induction of miR-200 as *Zeb1* alleles were mutated (Figure 4B), suggesting that it is induction of miR-200 when Zeb1 is mutated that is causing induction of Ets1. It is of note that further down-regulation of Zeb1 in *Zeb1* null MEF cells had little effects on miR-200 expression (Figure 4B), possibly due to [1] the level of Zeb1 protein in het MEF cells is low enough to relieve the repression on miR-200 family, further decrease in Zeb1 expression in null cells will not increase in miR-200 expression accordingly or/and [2] it is also possible that Zeb1 might down-regulate other unknown factor(s) that positively regulates expression of miR-200 family at the same time and thereby makes the regulation network more complex. Taken together, these results suggest an amplification loop between Ets1 and Zeb1, where Ets1 increases transcription of *Zeb1*, and Zeb1 in turn represses miR-200 to further elevate Ets1.

### Rb1 family repression of Zeb1 is a major component of inhibition of Ets1 by Rb1

Even though an Rb1-E2F complex binds to the *Ets1* promoter to reduce its expression, it is of note that Rb1 also represses Zeb1 [40], leading us to ask whether Rb1 repression of Zeb1 was also contributing to the Rb1-dependent downregulation of Ets1. As we demonstrated previously [16], mutation of Rb1 family members led to induction of Zeb1 and this induction of Zeb1 was accompanied by repression of miR-200 (Figure 4C) and, as shown above, Ets1 (Figure 1). Knockdown of Zeb1 in the TKO MEFs using shRNA lenvirirus, as described previously [17], eliminated much of the induction of Ets1 (Figure 4D), suggesting that induction of Zeb1 and repression of miR-200 as *Rb1* family members are mutated is contributing significantly to the upregulation of Ets1. These results suggest that Rb1-E2F binds the *Ets1* promoter to limit its level of expression, but it is induction of Zeb1 and in turn repression of miR-200 when the Rb1 family activity is lost that is responsible for most of the induction of Ets1.

### Ets1 and Zeb1 are expressed at the invasive front of K-Ras initiated mouse lung adenocarcinoma

**Figure 5:**
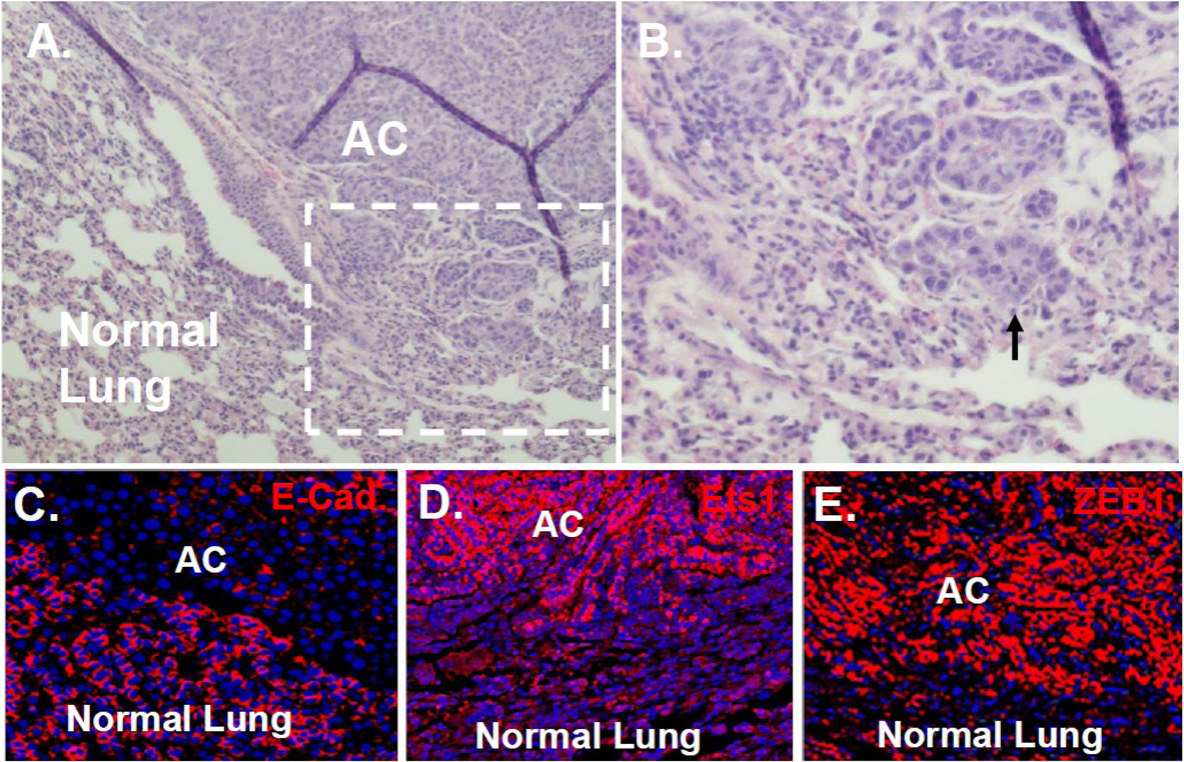
**Ets1 and Zeb1 are expressed at the invasive front of K-Ras initiated mouse lung adenocarcinoma. (A and B)** H&E staining showing the invasive front of an adenocarcinoma (AC) from a K-Ras^LA1^ mouse lung. **(C-E)** Note immunostaining for E-cadherin (E-cad.) in normal lung and expression of Zeb1 and Est1 in carcinoma invading into normal lung. Arrows indicate invasive carcinoma.

Ras pathway signaling causes Erk-dependent activating phosphorylation of Ets1 [13] and the resulting induction of Zeb1 is thought to be an important factor in Ras-initiated EMT (which is opposed by Rb1) [9]. Induction of Zeb1 leads to transition to a migratory mesenchymal phenotype and can been seen at the invading front of tumors [17]. We examine expression of Ets1 and Zeb1 in K-Ras initiated mouse lung adenocarcinomas (Figure 5A and B). Activation of an oncogenic K-Ras allele by recombination in somatic cells is sufficient for spontaneous lung cancer in K-Ras^LA1^ mice [34]. In this mouse model regions of atypical adenomatous hyperplasia (AAH) becomes evident in bronchial airways, and this hyperplasia expands to initiate formation of subpleural precancerous adenomas. Foci of adenocarcinoma *in situ* begin to appear within these precancerous lesions as the first evidence of transition to malignancy, and these adenocarcinomas subsequently expand and invade into surrounding lung tissue where E-cadherin is expressed (Figure 5C). In this model, Rb1 is a major barrier to tumor initiation, and it is inactivated by cdk hyperphosphorylation in these tumors [28]. Neither Ets1 nor Zeb1 were expressed in precancerous AAH or adenomas, but both were expressed at the invasive edge of adenocarcinomas (results not shown; Figure 5D and E).

### Correlation between Ets1 and Zeb1 in human lung adenocarcinomas

**Figure 6:**
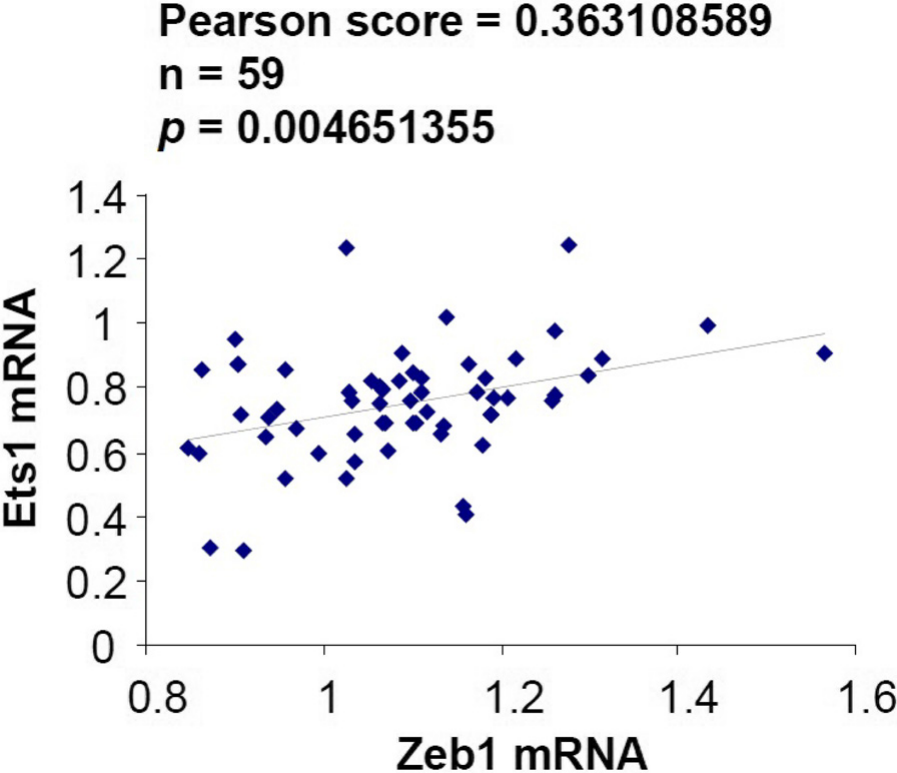
**Correlation between Ets1 and Zeb1 mRNA expression in human lung adenocarcinomas.** A Pearson plot is shown. See Methods for description of the microarray data base analysis.

To check if Ets1 also follows Zeb1 expression in human lung adenocarcinomas, we examined microarrays of 59 human lung adenocarcinomas [35] and compared expression of Ets1 and Zeb1 mRNAs. We found a significant correlation between expression of Ets1 and Zeb1 mRNA in these tumors (Figure 6). Taken together, these data with mouse and human lung adenocarcinomas are consistent with the amplification loop we described above for Ets1 and Zeb1.

### Mutation of Zeb1 eliminates Ets1 expression in differentiating thymocytes

**Figure 7:**
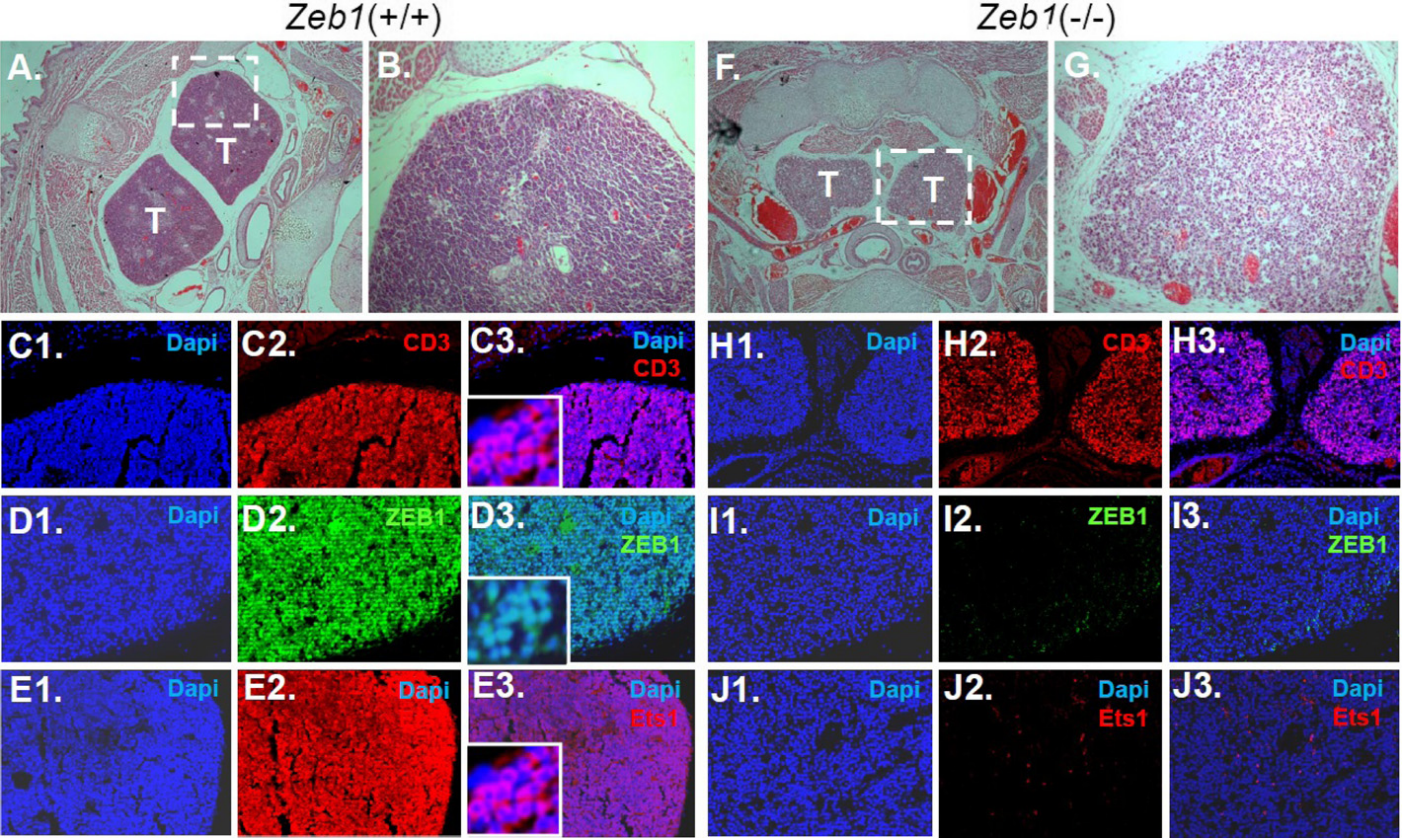
***Zeb1*** **mutation eliminates Ets1 expression in differentiating embryonic thymocytes. A**-**G** **H**&**E** sections of embryonic day 15.5 wild-type and *Zeb1*(−/−) embryos showing the thymus (T). Note the decrease in thymocyte number with *Zeb1* mutation. **(C-J)** Immunostaining showing loss of expression of Ets1 with Zeb1 mutation in differentiating thymocytes. CD3 is a T-cell marker.

Although the correlation between Ets1 and Zeb1 in lung adenocarcinoma seen above is consistent with an amplification loop between Ets1 and Zeb1, these results are also consistent simply with the previously documented induction of Zeb1 by Ets1 [9]. Therefore, we next asked whether Ets1 expression is dependent upon Zeb1. Mutation of Zeb1 and Ets1 both lead to defects in thymocyte differentiation [23-27]. *Zeb1*(−/−) mice show a decrease in the number of thymocytes (Figure 7A, B, C, F, G and H), and we found that this *Zeb1* mutation eliminated Ets1 expression in the thymocytes (Figure 7D, E, I and J). These results demonstrate that Ets1 expression in thymocytes is dependent upon Zeb1, and further confirm the linkage between the two transcription factors in the defects leading to thymocyte differentiation.

## Discussion

**Figure 8:**
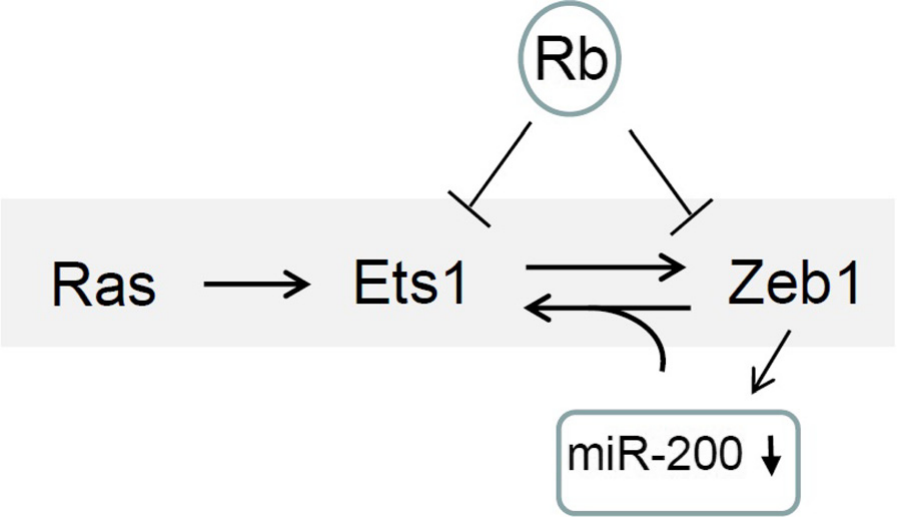
**Model depicting an Ets1-Zeb1 amplification loop that is dependent upon miR-200 and regulated by Rb1.** See text for discussion.

Mutation in the Ras pathway leading to growth factor independent signaling is frequent in tumors [11,12]. One consequence of Ras mutation is EMT, and this appears to be mediated at least in part by Erk phosphorylation and activation of Ets1 and in turn Ets1 induction of the EMT transcription factor Zeb1 [13]. The Ras pathway is under surveillance by the Rb1 tumor suppressor family, and mutation or inactivation of the Rb1 family is a hallmark of cancer required for progression of tumors with a Ras pathway mutation [22]. One consequence of Rb1 activation in response to Ras mutation is to suppress EMT. Our results suggest that E2F-Rb1 binds constitutively to the *Ets1* promoter and limits the level of expression, but a second major impact of Rb1 on Ets1 expression is mediated through Rb1 repression of Zeb1 [40] and in turn induction of miR-200, which targets Ets1. We propose that Ets1 and Zeb1 form an amplification loop that is dependent upon Zeb1 repression of miR-200 and regulated by the Rb1 family (Figure 8). We present evidence correlating expression of Ets1 and Zeb1 in invasive lung adenocarcinoma, suggesting that this amplification loop is evident in invasive lung cancer. Importantly, we demonstrate that Zeb1 is required for Ets1 expression in differentiating thymocytes providing evidence that Zeb1 is critical for Ets1 expression in vivo in the thymus. Beyond regulating expression of Zeb1 and EMT, Ets1 also induces expression of cyclin E and cdk2 to drive cell cycle progression [38]. Rb1 pathway mutation or inactivation is closely linked to unrestricted cell proliferation [22], and our results suggest that one component of this unrestricted cell cycle progression might be upregulation of Zeb1 and in turn Ets1 when the Rb1 pathway is lost. Interestingly, two separate transcriptional functions have been ascribed to Zeb1. Zeb1 binds to DNA through a series of zinc finger domains, and a C-terminal domain binds to the co-repressor CtBP to repress transcription [42]. The N-terminal domain of Zeb1 binds Smads and the transcriptional co-activator p300, and Zeb1 acts as a scaffold for assembly of an active smad-p300 complex at promoters of TGF and BMP-responsive genes [43]. Two different mutations of Zeb1 have been examined—a gene knockout and a mutation that deletes the N-terminal smad-p300 binding domain [23,30]. Knockout of Zeb1 leads to lethality at the end of gestation, with cleft palate, skeletal defects, disrupted epithelial vs. mesenchymal balance and diminished thymocyte differentiation [23]. Interestingly, deletion of the N- terminal domain of Zeb1 eliminates cleft palate and other skeletal defects, but the thymocyte differentiation defect persists [30]. These results imply that CtBP-dependent transcription repression of miR-200 family members by Zeb1 is important for regulation of Ets1 and for thymocyte differentiation.

## Conclusions

Our results provide evidence of an Rb1-dependent Ets1-Zeb1 amplification loop that is important in both cancer and in normal development of the thymus.

## Additional files

Additional file 1:
**Ets1 paper supplementary table 1, 7 K** **http://www.biomedcentral.com/imedia/8917713331383367/supp1.pdf.**
Additional file 2:
**Ets1 paper supplementary table 2, 10 K** **http://www.biomedcentral.com/imedia/7163581351383367/supp2.pdf.**
