# Supplementary material for: An Rb1-dependent amplification loop between Ets1 and Zeb1 is evident in thymocyte differentiation and invasive lung adenocarcinoma
Source: BMC Mol Biol. 2015 Mar 19;16:8. doi: 10.1186/s12867-015-0038-4 (PMC4364651; doi:10.1186/s12867-015-0038-4)
Supplement: Additional file 1: — Ets1 paper supplementary table 1, 7 K http://www.biomedcentral.com/imedia/8917713331383367/supp1.pdf. [file 12867_2015_38_MOESM1_ESM.pdf]

Supplementary table 1. Primary antibodies used for Immunohistochemistry

Name	IgG	Specificity	Manufacturer	Dilution
Anti-E-cadherin (E-cad)	mouse monoclonal	m, rat, h, dog	BD Biosciences	1:50
Anti-Zeb1	rabbit polyclonal	m	Dr. Doug Darling	1:200
Anti-Ets1	rabbit polyclonal	m, rat, h	Santa Cruz Biotechnology	1:200
Anti-CD3	rabbit polyclonal	m, rat, h	Daka (Dr. Qingxian Lu)	1:50-100
Anti-Rb1	rabbit polyclonal	m, rat, h	Santa Cruz Biotechnology	1:200
Anti-whole rabbit IgG	Sheep Ab-Cy3		Sigma	1:500
Anti-whole mouse IgG	Goat Ab-Alexa Fluor® 488		Molecular Probes	1:500
