# Supplementary material for: An Rb1-dependent amplification loop between Ets1 and Zeb1 is evident in thymocyte differentiation and invasive lung adenocarcinoma
Source: BMC Mol Biol. 2015 Mar 19;16:8. doi: 10.1186/s12867-015-0038-4 (PMC4364651; doi:10.1186/s12867-015-0038-4)
Supplement: Additional file 2: — Ets1 paper supplementary table 2, 10 K http://www.biomedcentral.com/imedia/7163581351383367/supp2.pdf. [file 12867_2015_38_MOESM2_ESM.pdf]

Supplementary table 2. Primers used for real-time PCR, microRNA detection and ChIP assay

Primer name	Sequence	Tm ˚C	Amplicon (bp)
Hs RRM2 LP	5'- GAAGGCAGAGGCTTCCTTTT	56.3	200
Hs RRM2 RP	5'- AGAAACAGCGGGCTTCTGTA	54.7	
Hs NTF3 LP	5'- CAGGTAGCTGGTGCCAGAAT	55.3	200
Hs NTF3 RP	5'- TGAGGGAATTGAGCGAGTCT	57.1	
Hs Ets1 LP	5'- TGGAGTCAACCCAGCCTATC	56.4	233
Hs Ets1 RP	5'- TCTGCAAGGTGTCTGTCTGG	57	
Mm Ets1 LP	5'- CCCAGAATCCTGTTACACCTCG	57.2	236
Mm Ets1 RP	5'- GCTTGATGGCAAAGTAGTCTGT	55.3	
Mm Zeb1 LP	5'- TGGCAAGACAACGTGAAAGA	60	200
Mm Zeb1 RP	5'- AACTGGGAAAATGCATCTGG	60	
Mm ACTB LP	5'- GGCTGTATTCCCCTCCATCG	57.6	154
Mm ACTB RP	5'- CCAGTTGGTAACAATGCCATGT	55.9	
Mm GAPDH LP	5'- AGGTCGGTGTGAACGGATTTG	57.6	123
Mm GAPDH RP	5'- TGTAGACCATGTAGTTGAGGTCA	55.1	
Mm mir200c LP	5'- taatactgccgggtaatgatgga	55.6	68
Mm mir200b LP	5'- taatactgcctggtaatgatga	51	68
Mm mir200a LP	5'- taacactgtctggtaacgatgt	53.5	68
Mm mir141 LP	5'- aacactgtctggtaaagatgga	53.6	68
Mm mir429 LP	5'- TAATACTGTCTGGTAATGCCGTAA	53.6	68
Mm miACTB LP	5'- GGCTGGCCTGTACACTGACTTGA	61.1	110
Mm miGAPDH LP	5'- CACTGAGCATCTCCCTCACA	56.6	155
Universal miR RP	5'- GCGAGCACAGAATTAATACGAC	53.9	
Mm Ets1 Promoter LP	5'- TCATCTGATGCGATCTGAGC	54.6	171
Mm Ets1 Promoter RP	5'- GGGCTGTCTCTAGCAGGATG	57.2	
